# Use of internet in adolescents and young adults with JIA

**DOI:** 10.1186/1546-0096-12-S1-P302

**Published:** 2014-09-17

**Authors:** Philomine Van Pelt, Constance Drossaert, Aike Kruize, Jaap Huisman, Radboud Dolhain, Nico Wulffraat

**Affiliations:** 1 pediatric immunology and rheumatology, Wilhemina's Children Hospital UMC Utrecht, Utrecht, Netherlands; 2 Rheumatology and pediatric rheumatology, ErasmusMC University, Rotterdam, Netherlands; 3 Department of Psychology, Health and Technology, University of Twente, Enschede, Netherlands; 4 Rheumatology and immunology, UMCU, Netherlands; 5 Pediatric Psychology, Wilhelmina's Children Hospital UMCU, Utrecht, Netherlands; 6 Rheumatology, ErasmusMC , Rotterdam, Netherlands; 7 Pediatric Immunology and Rheumatology, Wilhelmina's Children Hospital UMCU, Utrecht, Netherlands

## Introduction

Internet-use is increasing since it is an efficient way to find information. Information obtained via Health Related Internet (HRI) sites, or online peer support groups might increase knowledge and self-management in adolescents and young adults with Juvenile Idiopathic Arthritis (JIA).

## Objectives

To evaluate the frequency of use and perceived relevance of HRI and its association with demographic, disease-related and psycho-social variables

## Methods

In a cross-sectional study, JIA patients (age 10 - 27 years) were asked to complete a self-reported questionnaire. Frequency of using HRI-sites (regarding information about JIA, medication-use and aspects of JIA related to social life) as well as having online contact with fellow patients were evaluated. Perceived relevance of HRI and contact with fellow patients were also questioned. Demographic variables, disease activity, medication and emotional behavior and coping were assessed as possible predictors.

## Results

142 patients were included. 71% had used internet to search general information on JIA, but specific topics like medication, were less searched for. 25% had ever visited a forum or had online peer contact. Perceived relevance of HRI-sites and opportunities for online peer contact was rated low (median 2.0; scale 0-10). Demographic and disease related factors, apart from female gender, were not associated with HRI use. Among psychological variables, in male, only coping styles “confrontation” and “reassuring thoughts” were associated with HRI use. Emotional behavior was not associated.

## Conclusion

Health Related Internet cannot be used as the only source of information for JIA patients since they use it infrequent and consider its relevance low.

## Disclosure of interest

None declared.

